# Operational utility of the reverse-transcription recombinase polymerase amplification for detection of dengue virus

**DOI:** 10.1186/s12879-018-3065-1

**Published:** 2018-04-11

**Authors:** Kim-Kee Tan, Noor Syahida Azizan, Che Norainon Yaacob, Nurul Asma Anati Che Mat Seri, Nur Izyan Samsudin, Boon-Teong Teoh, Sing-Sin Sam, Sazaly AbuBakar

**Affiliations:** 1 Tropical Infectious Diseases Research and Education Centre (TIDREC), University of Malaya, 50603 Kuala Lumpur, Malaysia; 2 WHO Collaborating Centre for Arbovirus Research and Reference (Dengue and Severe Dengue), University of Malaya, 50603 Kuala Lumpur, Malaysia; 3 Department of Medical Microbiology, Faculty of Medicine, University of Malaya, 50603 Kuala Lumpur, Malaysia

**Keywords:** Infectious diseases, Diagnostics, Dengue, Isothermal, PCR, RT-RPA

## Abstract

**Background:**

A method for rapid detection of dengue virus using the reverse-transcription recombinase polymerase amplification (RT-RPA) was recently developed, evaluated and made ready for deployment. However, reliance solely on the evaluation performed by experienced researchers in a well-structured and well-equipped reference laboratory may overlook the potential intrinsic problems that may arise during deployment of the assay into new application sites, especially for users unfamiliar with the test. Appropriate assessment of this newly developed assay by users who are unfamiliar with the assay is, therefore, vital.

**Methods:**

An operational utility test to elucidate the efficiency and effectiveness of the dengue RT-RPA assay was conducted among a group of researchers new to the assay. Nineteen volunteer researchers with different research experience were recruited. The participants performed the RT-RPA assay and interpreted the test results according to the protocol provided. Deviation from the protocol was identified and tabulated by trained facilitators. Post-test questionnaires were conducted to determine the user satisfaction and acceptability of the dengue RT-RPA assay.

**Results:**

All the participants completed the test and successfully interpreted the results according to the provided instructions, regardless of their research experience. Of the 19 participants, three (15.8%) performed the assay with no deviations and 16 (84.2%) performed the assay with only 1 to 5 deviations. The number of deviations from protocol, however, was not correlated with the user laboratory experience. The accuracy of the results was also not affected by user laboratory experience. The concordance of the assay results against that of the expected was at 89.3%. The user satisfaction towards the RT-RPA protocol and interpretation of results was 90% and 100%, respectively.

**Conclusions:**

The dengue RT-RPA assay can be successfully performed by simply following the provided written instructions. Deviations from the written protocols did not adversely affect the outcome of the assay. These suggest that the RT-RPA assay is indeed a simple, robust and efficient laboratory method for detection of dengue virus. Furthermore, high new user acceptance of the RT-RPA assay suggests that this assay could be successfully deployed into new laboratories where RT-RPA was not previously performed.

**Electronic supplementary material:**

The online version of this article (10.1186/s12879-018-3065-1) contains supplementary material, which is available to authorized users.

## Background

Early diagnosis of dengue depends on the detection of the virus by either nucleic acid amplification test (NAAT) [1–4] or detection of dengue virus (DENV) nonstructural protein 1 (NS1) antigen especially that configured into the rapid detection test (RDT) format [5–9]. The NS1 detection method is among the most widely used as it is rapid and simple to perform [9]. The method, however, has its limitation, especially when utilized in dengue endemic regions where secondary dengue is common [5, 9–11]. NS1 assay sensitivity in detection of secondary dengue infection is much lower, hence, may contribute to false negative results [12, 13]. A complementary detection method is therefore needed [14]. The NAAT has been suggested as the most suitable complementary test since the test allows for direct detection of DENV genome from samples of patients obtained during the viremic phase (< 5 days after fever onset). While there are a number of NAATs available, the most common NAAT method for detection of DENV has been the quantitative reverse-transcription polymerase chain reaction (RT-PCR) [15–17]. The test is highly sensitive, specific, and can be easy to perform especially by trained personnel. Unfortunately, due to its requirement for highly specific equipment and reagents, usage of the test has been confined to the well-funded and well-equipped referral laboratories [18, 19]. The use of NAAT in a resource-limited setting such as peripheral laboratories in many dengue endemic regions of the Southeast Asia is, therefore still limited [20–22]. In recent years, extensive efforts have been undertaken to develop NAAT for the use in these resource-limited settings for various infectious diseases [23–26]. Implementation of the NAAT as a preferred diagnostic test in this setting, however, remained challenging. The ideal diagnostic test should be a simple, rapid, sensitive, specific, and requires minimal laboratory infrastructure [27]. A more cost-effective NAAT format that met all the aforementioned criteria hence, is needed.

The invention of the isothermal NAAT that requires no or minimum laboratory infrastructure has the potential to overcome the barrier to the use of NAAT in resource-limited setting [28–31]. The isothermal NAAT usually has a simple protocol, easy to perform, does not require a sophisticated instrument, and straightforward result interpretation procedure [32–34]. We recently reported a simple, rapid, sensitive, and specific single tube pan-dengue reverse-transcription recombinase polymerase amplification (RT-RPA) method for early detection of DENV [1]. This method showed comparable sensitivity to the reference real-time RT-PCR test. The dengue RT-RPA assay was performed on an inexpensive portable fluorometer and took only approximately 20 min to perform with minimal reagent and equipment cost. This NAAT assay hence, has a potential for use in a resource-limited setting [1]. However, a diagnostic assay with excellent performance in itself is insufficient. The feasibility of a diagnostic test for use in resource-limited setting relies heavily on the robustness of the assay and acceptability by the end users. With this in mind, in the present study, we assessed the operational utilities of the previously described dengue RT-RPA assay, which includes ease to use, the time required to perform the assay, and user acceptability.

## Methods

### Study design

The usability of performing the RT-RPA assay was assessed among a group of volunteers who were new to the assay. The operational usability of the RT-RPA was evaluated for three focus areas: 1) effectiveness, 2) efficiency and 3) satisfaction. The definitions of the terms of the three areas were as defined below:

1. Effectiveness: the accuracy and completeness with which users can achieve specified goals in a particular environment.
2. Efficiency: the time needed to complete the tasks strictly according to the provided written protocols.
3. Satisfaction: the comfort and acceptability of the work system to the users.

### Participants

Seven researchers were recruited from the Arbovirus Surveillance Laboratory at the Tropical Infectious Disease Research and Education Centre (TIDREC). In addition, we recruited two groups of researchers from i) molecular research laboratories (7 researchers) and ii) antiviral research laboratory (5 researchers) from the Department of Medical Microbiology, Faculty of Medicine, University of Malaya (UM), to participate in the usability testing. A total of 19 researchers with different research experience participated in the study (Additional file 1: Table S1).

### Reagent and DENV RNA

The DENV-specific TwistAmp RT *exo* lyophilized kit was supplied by TwistDx Ltd., Cambridge, United Kingdom under the European Union FP7 DengueTools agreement 282589 [1]. The lyophilized kit consisting of 1) lyophilized RT-RPA pellet with dengue-specific primers, probes, and fluorescently-tag probes, and 2) customized rehydration buffers consisted of magnesium acetate, potassium acetate, Tris-acetate, and polyethylene glycol 35,000. A random subset of 152 RNA samples extracted from the sera of dengue-suspected patients received between March and May 2015 were used. DENV RNA samples were provided by the WHO Collaborating Centre for Arbovirus Reference & Research (Dengue/Severe Dengue) (WHO CC) at the UM. The reference RT-RPA assay was initially performed by trained laboratory personnel of the WHO CC. The WHO CC at UM had previously successfully participated in the WHO WPRO-conducted EQA program for dengue diagnostics [19]. Results from the reference RT-RPA assay were used as the reference for the current study. Out of the 152 RNA samples, 97 were dengue RT-RPA positive, and 55 were dengue RT-RPA negative samples. In the WHO CC laboratory, all the dengue RT-RPA positive samples showed positive amplification results between 5 and 8 min after initiating the assay. From these samples, each participant was randomly provided with eight samples to test.

### Setting

**Fig. 1:**
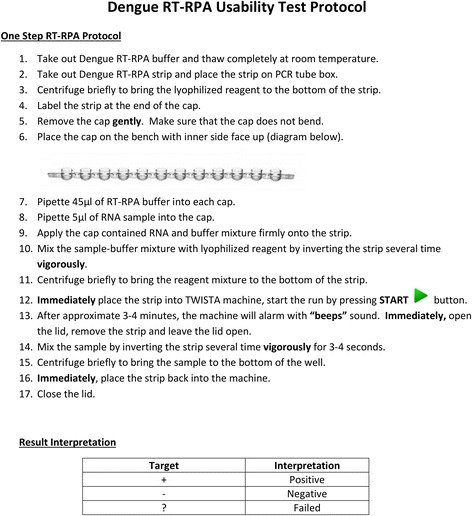
Dengue RT-RPA usability test protocol

The participants were requested to perform the RT-RPA assay following a given standard written protocol (Fig. 1). The participants conducted the assay individually by reading the protocol, and there was no discussion among the participants. During the test, each participant was accompanied by one test facilitator who observed and took notes of the user’s conduct of the test. After the run, participants were asked to record the test results. Following completion of the test, participants were requested to complete a post-testing questionnaire adapted from the After-Scenario Questionnaire developed by Lewis [35] to gauge the user’s acceptance and satisfaction towards the test.

### Statistical analysis

Statistical analysis was performed using Prism GraphPad V5.01 [36]. One-way ANOVA was performed to determine the mean difference between the participants with different research experience against the number of deviations from the standard protocol. The same statistical test was also employed to assess the mean difference of groups with 1) different number of deviations from the standard protocol, and 2) different years of research experience against the result accuracy obtained.

## Results

### Ease of performing the improved RT-RPA assay

Nineteen participants who had never performed RPA assay were enrolled into this usability testing. The participants were grouped according to their respective laboratories and research experience. Based on the participant’s research experience, 31.6% (6/19) of the participants were with less than 1-year of research experience, 36.8% (7/19) with 1 to 5 years of research experience, while the remaining 31.6% (6/19) of the participants had more than 5 years of research experience. All 19 participants completed the test.

### Effectiveness

The effectiveness of the given instruction as the standard protocol to perform the test was evaluated for completeness and accuracy with which the user performed the test (Table 1). During the usability test, of the 19 participants, 15.8% of the participants performed the test without deviations. The majority of the participants (84.2%) performed the test with 1 to 5 deviations from the protocol. None of the participants had more than 5 deviations from the protocol during the test.

**Table 1:** Number of deviations from the protocol during the test

Details	Number (%) of participant who performed the test correctly
Number of participants who performed the test with deviations from protocol	
a. 0 deviation	3 (15.8%)
b. 1 to 5 deviations	16 (84.2%)
c. More than 5 deviations	0 (0%)

In order to investigate the possible correlation between the participants’ research experience and the deviations that occurred during the test, we analyzed the deviations from the protocol and their respective years of research experience (Table 2). For participants with research experience of less than 1 year, 2 out of 6 participants (33.3%) performed the test without deviations, while 66.7% of the participants with less than 1-year research experience performed the test with 1 to 5 deviations. None of the participants from the group with less than 1-year research experience performed the test with more than 5 deviations. For the group of participants with 1 to 5 years research experience, all of them (100%) performed the test with 1 to 5 deviations from the protocol. For participants with more than 5-year research experience, only 1 participant (16.7%) performed the test without deviations. The remaining of the participants (83.3%) performed the assay with 1 to 5 deviations. There were no statistically significant (*p*-value = 0.5545; one-way ANOVA; Table 3) differences of the mean of deviations occurred during the RT-RPA procedure between the groups of participants with a different year of research experience (< 1 year, 1 to 5 years, and > 5 years).

**Table 2:** Number of deviations from protocol during the test for participant with different research experience

Details	Number (%) of participant who perform the test correctly
Number of participants with research experience less than 1 year who performed the test with deviations from the protocol	
a) 0 deviation	2 (33.3%)
b) 1 to 5 deviations	4 (66.7%)
c) More than 5 deviations	0 (0%)
Number of participants with research experience of 1 to 5 years who performed the test with deviations from the protocol	
a) 0 deviation	0 (0%)
b) 1 to 5 deviations	7 (100%)
c) More than 5 deviations	0 (0%)
Number of participant with research experience more than 5 years who perform the test with deviations from the protocol	
a) 0 deviations	1 (16.7%)
b) 1 to 5 deviations	5 (83.3%)
c) More than 5 deviations	0 (0%)

**Table 3:** Summary of One-way ANOVA analysis

Type of analysis	*p*-value	Significance (*P* < 0.05)
Research experience versus number of deviations	0.5545	No
Research experience versus result accuracy	0.1715	No
Number of deviation versus result accuracy	0.3369	No

We analyzed the number of deviations that occurred for each step during the test (Table 4). Results obtained suggested that step 10 and 14 that involved “inverting the strip several times vigorously” showed the highest percentage of deviations from the protocol. 14 out of 19 participants (73.7%) performed step 10 with deviations. For step 14, 9 out 19 participants (47.4%) performed this step with deviations. 3 out of 19 participants (15.8%) performed step 13 which involved removing the test strip from the machine after the machine alarm gave “beep” sound, with deviations.

**Table 4:** Number of deviations of each step during the one-step RT-RPA experiment

Step	Deviations	
	Number	Percentage (%)
1	0	0.0
2	1	5.3
3	2	10.5
4	1	5.3
5	1	5.3
6	1	5.3
7	2	10.5
8	2	10.5
9	2	10.5
10	14	73.7
11	0	0.0
12	3	15.8
13	3	15.8
14	9	47.4
15	0	0.0
16	2	10.5
17	0	0.0

Out of 152 samples, we were unable to detect the signal (positive or negative) for two samples (Additional file 2: Table S2). These samples were considered to have failed the RT-RPA assay. These samples were tested by the same participant, suggesting that the failure could be operator-dependent. The overall accuracy of the RT-RPA assay was calculated based on the remaining 150 samples. By comparing the test results of the test performed by the participants against the reference test results, the accuracy of test performed by the participants was at 89.3% (134 /150; Table 5). Of the 16 samples that contributed inconsistent results, seven were false positive, and nine were the false negative. Two out of the false positive samples displayed negative amplification pattern on the read-out graphs. These two negative samples somehow were called as ‘positive’ with the built-in software in Twista® fluorometer (TwistDx, UK). While for the false negative samples, eight out of nine showed late amplification pattern, suggesting low target copy number RNA samples.

**Table 5:** Comparison of the RT-RPA results obtained by the participant against the reference test results

Reference	Participant’s RT-RPA test result	
	Positive	Negative
Positive	87	9
Negative	7	47

The One-way ANOVA analysis was also used to investigate the relationship between the number of the deviations from the protocol, and participant’s research experience versus the result accuracy. Our results showed that there were no statistically significant mean differences in the result accuracy between groups of participants with different research experience (Table 3). Similarly, the number of deviations from the standard protocol did not significantly affect the result accuracy (Table 3).

### Efficiency

The efficiency of performing the test was evaluated by the time needed for the participants to complete the test for all eight samples. This included active working time, 15 min amplification time, and time required for interpretation of the results. In the present study, all participants completed the test within 30–45 min.

### Satisfaction

**Fig. 2:**
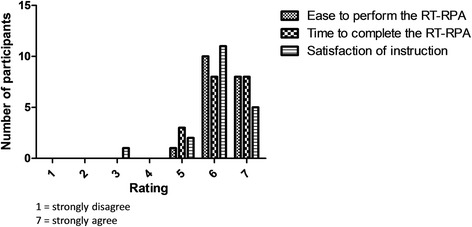
User satisfaction in performing the one-step RT-RPA. The user satisfaction on one-step RT-RPA assay was evaluated based on three categories; 1) ease to perform the RT-RPA assay, 2) time that needed to complete the RT-RPA assay, and 3) satisfaction of the given written instruction on RT-RPA. The user satisfaction for each category was rated by a scale of 1 to 7 (strongly disagree to strongly agree)

The overall satisfaction of test participants toward the RT-RPA test was assessed by using the post-test questionnaire. The assessment was divided into two parts; the participants were asked to evaluate the satisfaction for i) performing the one-step RT-RPA protocol and ii) interpreting of results. By using 7-point Likert scales, the average score for performing the one-step RT-RPA protocol was 6.21 (Fig. 2; Additional file 3: Table S3). Overall, as high as 90% of the participants gave positive feedback. Satisfaction for ease of performing the test and time needed to perform were 100%. One participant was not satisfied (rating = 3) with the support documentation supplied with the test.

**Fig. 3:**
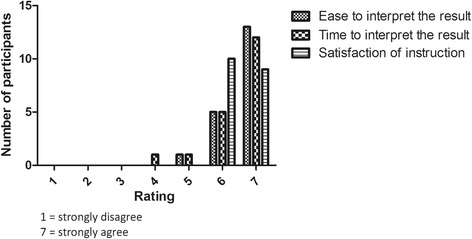
User satisfaction in the interpretation of results. The user satisfaction on one-step RT-RPA assay was evaluated based on three categories; 1) ease to interpret result, 2) time that needed to interpret result, and 3) satisfaction of the given written instruction on result interpretation. The user satisfaction for each category was rated by a scale of 1 to 7 (strongly disagree to strongly agree)

For interpretation of results, the average score was 6.53, which suggested high positive feedback from the participants (Fig. 3; Additional file 4: Table S4). Satisfaction for the ease to interpret the results and support documentation provided for the test were 100%. One participant gave a score of 4 (moderate) for satisfaction in the time required to interpret the results.

## Discussion

This study represents an analysis of the operational utility of the DENV nucleic acid detection method, RT-RPA in preparation for its possible deployment as a laboratory diagnostic tool. Implementation of a diagnostic test in a new testing environment is highly challenging, particularly in a resource-limited environment. The RT-RPA assay was previously described as the most rapid molecular diagnostic tools for detection of DENV [1], but these studies were conducted under laboratory conditions. A well-designed assay, however, does not necessarily define the successfulness of the test adoption in a new environment [37]. In the real world context, laboratory setting or field-testing site environment can be highly variable in its infrastructure, availability of instrumentation, and level of experience of the laboratory personnel. The best-developed test may not have a significant impact unless it is well-adapted into the existing diagnostic system and can be properly performed. The challenges are further compounded if it is to be performed in a resource-limited environment where the laboratory infrastructure could be limited. Dengue, in particular, is endemic in many economically developing regions where resources for proper laboratory testing can be scarce outside the major referral healthcare facilities often in the much urbanized capital cities. The operational utility evaluation of the RT-RPA assay prior to its deployment and implementation, hence greatly increase the chances of its successful adoption. The operational utility evaluation would also allow for the necessary adjustments before the assay deployment.

The dengue RT-RPA assay is easy to perform and does not require specialized equipment and high level of skill [38]. In addition to its potential benefit for use in a resource-limited setting, the test could be implemented and adopted into the diagnostic algorithm of a well-established laboratory including those at the referral level [1, 39]. With this in mind, in the study, laboratory personnel with different research experience were recruited from different laboratories. This is important to strengthen the representativeness of the operational utility test. We found no significant differences, however, in the number of deviations from the RT-RPA protocol to the research experience of the participants and their laboratory background in performing the RT-RPA assay. The deviations from the protocol may be random and operator-dependent. In addition to that, we also found that the number of deviations from the protocol showed no significant influences on the result accuracy. The differences in result accuracy were probably linked to the specific technical procedure requiring a mixing step for the redistribution of the amplicon aggregates formed during the amplification. In the present study, we used the Twista® fluorometer (TwistDX, UK) to read the amplification. At about 3 min and 45 s after initiation of the test, a beeping alarm would remind the user to remove the tube strip from the fluorometer. Sample mixing was done manually by the operator according to the written instruction [40]. It was at this specific step in the testing process that tendency for deviation was high, especially among new RT-RPA users or in the field setting [41]. This was confirmed in our study, as there were high deviations among users who performed the mixing step, including the way they mixed the sample, the duration of the mixing and the extent of vigor exerted during the mixing, regardless of their research experience. The main reason for these deviations is probably that this specific step is usually not employed in other NAATs. The manual mixing procedure, however, is needed to overcome potential localized depletion of reagents in the area of high RT-RPA activity on the nucleic acid template within the reaction tube [40]. This is a critical step in the amplification cascade to ensure the continuation of the RT-RPA assay and efficient consumption of the reaction mix. In all RT-RPA assays, the mixing step is highly recommended, especially for samples with a low copy number of the target [41]. Obviously, this is the inherent limiting step of the RT-RPA assay needing further attention by the assay developer.

Simplification or elimination of the manual mixing step may help reduce the inter-user variability. One possibility to rid of the manual mixing step is to perform RT-RPA reaction in low volume (5 μl) [41]. However, this may not be feasible as at the onset of fever in dengue; there could be as high as 1 X 10^8^ of DENV particles per milliliter of blood [42]. The high concentration of the RNA targets has been shown to affect the RPA assay performance adversely [43]. A recent development of the latest RT-RPA fluorometer, the T-8 isothermal device (TwistDX, UK) that included a built-in magnetic mixing function may obviate the need for the manual mixing [41], and this could help to overcome the current limitation of the assay. With this improvement, the effectiveness of the assay should increase, and the inconsistency due to operator-related factors would be reduced.

User satisfaction is the user’s feeling whether the test is easy to perform, the result is easy to interpret, and their readiness to perform the test. The perspective from new users could provide an important comment on the robustness and feasibility of the assay in the actual user application. Generally, user satisfaction is interrelated with the ease of use and efficiency of the assay. An easy to use assay is usually well-accepted by the user. Results from our study suggested very high acceptability of the dengue RT-RPA assay among the new users. This was probably because i) the required reagents were already in lyophilized format, ii) only minimum pipetting steps were needed comparing to the standard RT-PCR or real-time RT-PCR method, and iii) the assay involved only short incubation time of less than 20 min compared to the standard RT-PCR (more than 3 h) and real-time RT-PCR (45 min to 2 h).

## Conclusion

In conclusion, the current study demonstrates the operational utility of the newly developed dengue RT-RPA assay. We showed that the dengue RT-RPA assay was robust, easy to use, efficient, and can easily be performed. Based on our finding, it is likely that the RT-RPA assay will receive high acceptance by most possible new users. Implementation of the dengue RT-RPA could be impactful especially in a resource-limited environment where dengue is endemic, and it could also be a useful complementary test in referral laboratories. Simplification of the RT-RPA protocol by integrating the built-in magnetic mixing function in place of the current manual step should be considered prior to the actual assay deployment.

## Additional files

Additional file 1: **Table S1.** Number of participants. (DOCX 44 kb)

Additional file 2: **Table S2.** Data on RT-RPA amplification. (XLSX 10 kb)

Additional file 3: **Table S3.** Data on user satisfaction in performing the one step RT-RPA. (XLSX 9 kb)

Additional file 4: **Table S4.** Data on user satisfaction in the interpretation of results. (XLSX 9 kb)

## Abbreviations

DENV: Dengue virus
NAAT: nucleic acid amplification test
NS1: nonstructural protein 1
RDT: Rapid detection test
RT-PCR: reverse-transcription polymerase chain reaction
RT-RPA: reverse-transcription recombinase polymerase amplification
UM: University of Malaya
WHO CC: WHO Collaborating Centre
