# Supplementary material for: Operational utility of the reverse-transcription recombinase polymerase amplification for detection of dengue virus
Source: BMC Infect Dis. 2018 Apr 11;18:169. doi: 10.1186/s12879-018-3065-1 (PMC5896040; doi:10.1186/s12879-018-3065-1)
Supplement: Supplementary file 1 — Table S1. Number of participants. (DOCX 44 kb) [file 12879_2018_3065_MOESM1_ESM.docx]

Supplementary Table 1. Number of participants

|  | **Number** |
| --- | --- |
| Number of participants | 19 |
| Number of participant with research experience |  |
| below 1 year  1 – 5 year(s)  More than 5 years | 6  7  6 |
| Number of participant with research experience |  |
| 1. Surveillance laboratory 2. Molecular research laboratory 3. Antiviral research laboratory | 7  7  5 |
| Number of participants who did not complete the test | 0 |
| Number of time that the test yielded no result | 0 |
| Number of participants who answered the post usability test | 19 |
